# Tibiofemoral rotation alignment in the normal knee joints among Chinese adults: a CT analysis

**DOI:** 10.1186/s12891-020-03300-7

**Published:** 2020-05-23

**Authors:** Yufeng Lu, Xiaoyu Ren, Benyin Liu, Peng Xu, Yangquan Hao

**Affiliations:** 1 Osteonecrosis and Joint Reconstruction Ward, Department of Joint Surgery, Honghui Hospital, Xi’an Jiaotong University, Xi’an, Shaanxi 710054 P.R. China; 2 Department of Radiology and Imaging Sciences, Honghui Hospital, Xi’an Jiaotong University, Xi’an, Shaanxi 710054 P.R. China

**Keywords:** Total knee arthroplasty, Rotational alignment, Tibial component, Anatomic landmarks, Reference lines, Reliability

## Abstract

**Background:**

Consensus on tibial rotation in total knee arthroplasty (TKA) remains controversial. The present study aimed to investigate the closest anatomical reference to surgical epicondylar axis (SEA) among 10 tibial markers in Chinese adults.

**Methods:**

This study included examination of 122 normal lower extremities. Briefly, 10 axes were drawn on the axial sections: transverse axis of tibia (TAT), axis of medial edge of patellar tendon (MEPT), axis of medial 1/3 of patellar tendon attachment (M1/3), Akagi line, Insall line, axis of medial border of tibial tubercle (MBTT), and axis of anterior border of the tibia 1–4 (ATC1–4). The mean angles between TAT and SEA and that between other axes and the line perpendicular to SEA were measured. Pairwise differences among the 10 tibial axes were examined by applying one-way analysis of variance (ANOVA) and paired t-test.

**Results:**

In all the knees, the mean angles of M1/3, Akagi line, Insall line, MBTT, ATC1, ATC2, ATC3, and ATC4 axes were compared to the line perpendicular to the projected SEA and found to be 10.2 ± 5.1°, 1.4 ± 5.0°, 11.9 ± 5.4°, 3.6 ± 4.8°, 12.0 ± 6.9°, 7.2 ± 8.6°, 7.1 ± 10.4°, and 6.6 ± 13.5° external rotation, respectively, and the MEPT axis was 1.6 ± 4.5° internal rotation. The mean angle for TAT was 4.1 ± 5.3° external rotation. The M1/3 and Insall line were significantly more externally rotated than Akagi line, MEPT, MBTT, TAT, ATC2, ATC3, and ATC4 axes. No significant differences were noted between the TAT axis and the MBTT axis and among the ATC2, ATC3, and ATC4 axes.

**Conclusion:**

The Akagi line, MBTT, and TAT showed good consistency with SEA in the axial femorotibial alignment with knee in extension. The middle segment of the anterior tibial crest also demonstrated good alignment consistency with SEA for the axial femorotibial alignment. Hence, these markers can be used as reliable references for rotational alignment of the tibial component in TKA.

## Background

Rotation alignment between the femoral and tibial components is extremely important for the success of total knee arthroplasty (TKA). Rotation malalignment can result in the development of symptoms such as anterior knee pain [1], poor patellar tracking [1, 2], instability of knee flexion [1, 3], and premature wear of polyethylene liner [1, 4].

The surgical epicondylar axis (SEA), which is the line connecting the tip of the lateral epicondyle to the medial epicondylar sulcus, has been shown to be a useful anatomic reference axis as well as a functional flexion–extension axis of the knee [5–9]. Therefore, SEA is well recognized as a reliable and stable anatomical reference marker for femoral rotation positioning in TKA. Theoretically, the line perpendicular to the projected SEA on the tibia can be considered as an effective reference for the rotation alignment of the tibial prosthesis in an extended knee [10–13]. However, this line cannot be directly marked intraoperatively, and some anatomical markers are essential to act as reference. For instance, the tibial posterior condylar line [14], the tibial transcondylar line [15], the line connecting the middle of the posterior cruciate ligament (PCL) insertion to the medial third of the tibial tubercle (Insall line) [16], the medial edge of the tubercle [17], the axis connecting the middle of the PCL insertion to the medial edge of the patellar tendon attachment (Akagi line) [11], medial sixth of the patellar tendon at the tibial attachment [18], the axis going from 1-mm medial of the tibial tubercle medial edge to that between the midsulcus of the tibial spines (as defined by Dalury) [19], the ankle transmalleolar axis [10], and the second metatarsal [10] are all popular tibial markers. However, the consensus on the application of these rotational alignment references of the tibial component remains controversial.

At our institution, the anterior tibial crest has often been used as the reference line for setting the tibial rotational orientation in TKA. Until date, only a few studies have investigated the accuracy and reliability of the anterior tibial crest as a rotational alignment reference for tibial components.

Through this study, we have attempted to determine 1) the reference line closest to SEA or to the line perpendicular to SEA from among the following 10 popular reference markers: Insall line, Akagi line, anterior tibial crest1–4, medial edge of patellar tendon, the transverse axis of the tibial (TAT) resected bone surface, medial 1/3 of patellar tendon attachment, and medial border of the tibial tubercle (MBTT) and 2) whether the anterior tibial crest can be used as a reliable anatomical reference for correcting the rotational alignment of the tibial components in TKA.

## Methods

Patients who underwent computed tomography angiography (CTA) examination of both the lower limbs due to trauma or tumor of the unilateral lower extremity at the Xi’an Honghui hospital between July 2017 and June 2018 were included in this study. All CT data were sourced from a digital image archive system (Picture Archiving and Communications Systems [PACS] Synapse, Fujifilm Inc., Tokyo Japan). The study protocol was approved by the hospital’s Ethics Committee. The inclusion criteria were as follows: 1) CT scan at a direction perpendicular to that of the lower limbs; 2) at least one side of the lower limb without fracture or tumor; 3) no obvious flexion, varus, and valgus deformity in the bilateral knees; and 4) no obvious degeneration in the bilateral knees. The exclusion criteria were as follows: 1) both the lower limbs with a fracture, residual internal fixation, tumor or any other pathological conditions; 2) three-dimensional (3D) reconstruction revealing incomplete extension of both the knee and hip joints; and 3) obvious deformity of the external tibial arch.

Transverse CT scans (SOMATOM Definition; Siemens Inc., Munich, Germany) were prepared with 1-mm thickness and at 1-mm interval, ranging from lumbar 4/5 intervertebral space to the sole of the foot, including the entire lower extremity. For scanning, the patient was asked to lay in a supine position with both the lower limbs straight.

All measurements were performed with PACS.

**Fig. 1:**
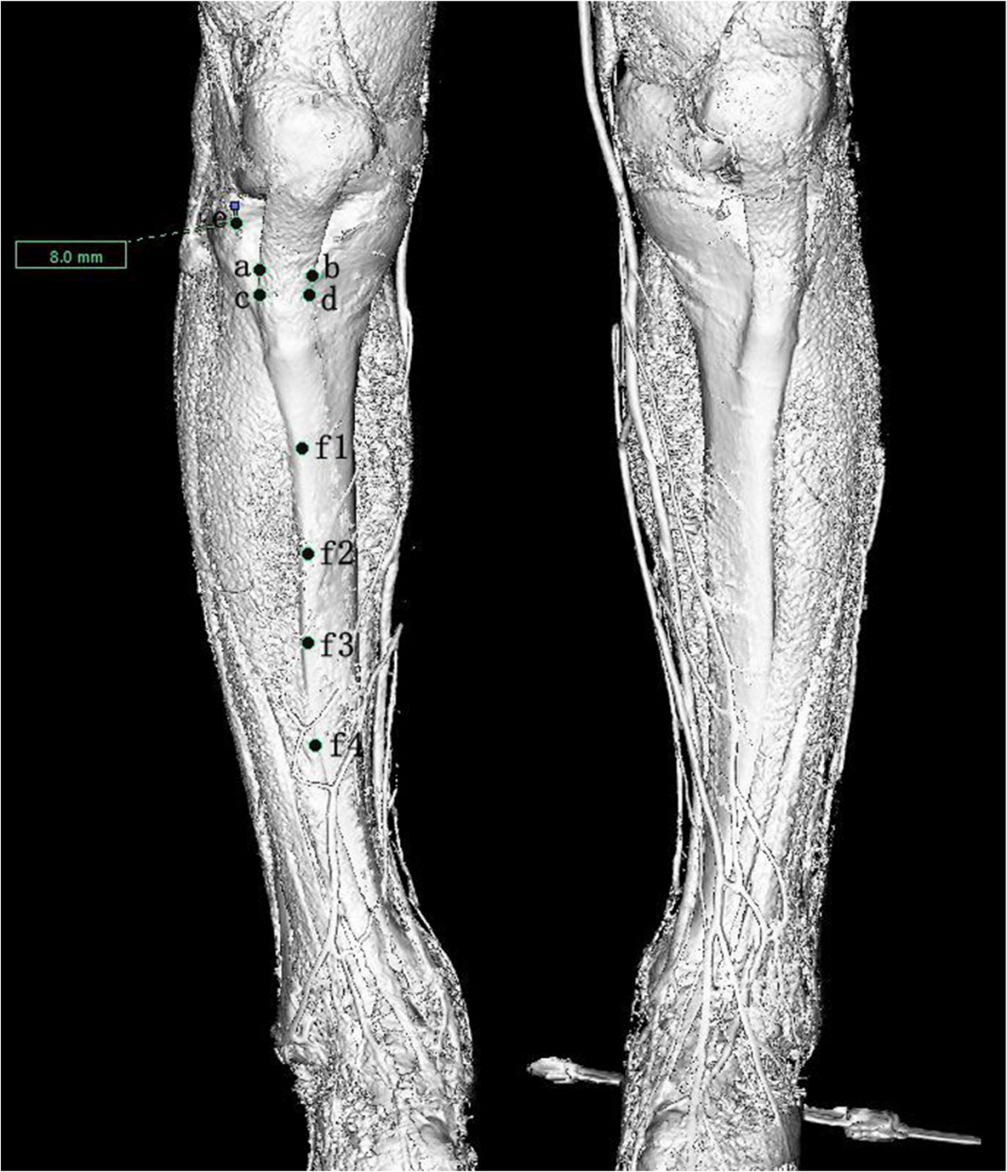
Landmarks at 3D reconstruction of the tibia: (1) the point (e) 8-mm distal to the lateral tibial plateau, (2) the medial (b) and lateral border (a) at the level of patellar tendon attachment, and (3) the medial (d) and lateral border (c) at the widest part of the tibial tubercle, (4) isometric marking of 4 points(f1,f2,f3,and f4) along the sharpest margin of the tibial anterior border

The 3D reconstruction of CT data was performed, and the marks were made as listed below: 1) the medial edge of a patellar tendon 8-mm distal to the lateral tibial plateau; 2) the medial and lateral border of the patellar tendon at the tibial attachment, connecting the 2 points; 3) the medial and lateral border of the widest part of the tibial tubercle, connecting the 2 points; 4) the proximal and distal ends of the sharp margin of the anterior tibial crest and 2 points between them on the anterior tibial crest to make a distance trisection (Fig. 1).

Finally, a total of 122 lower extremities (67 left and 55 right extremities) belonging to 122 patients were examined. The patients included 89 men and 33 women, with a mean age of 51.4 years (age range: 18–81 years).

**Fig. 2:**
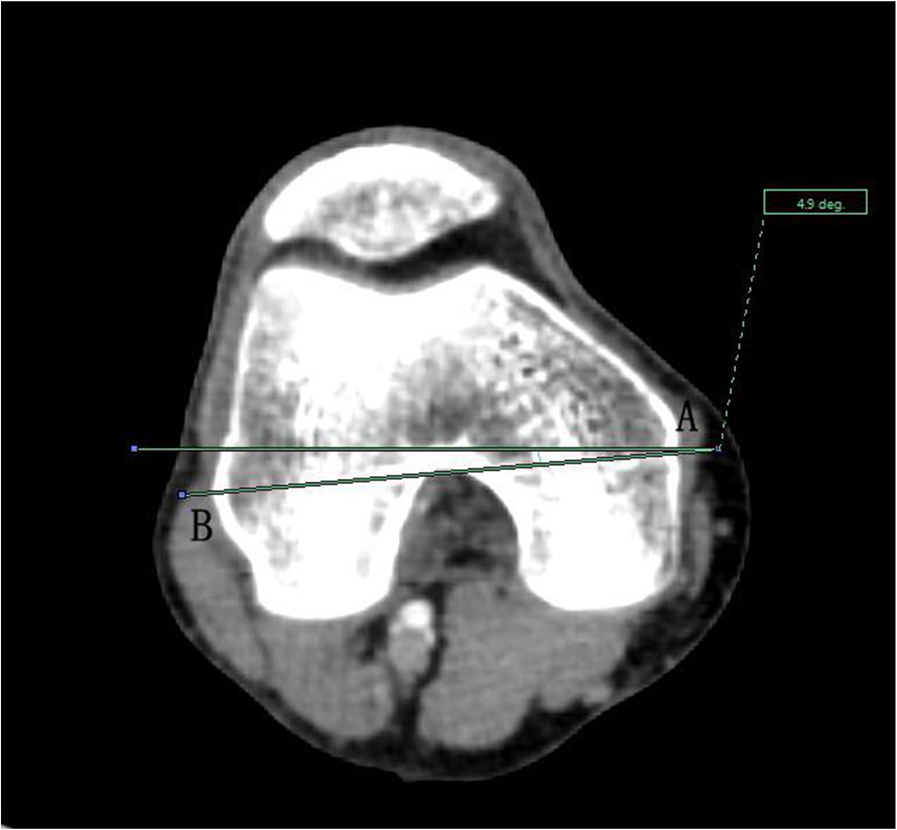
The surgical epicondylar axis (SEA) was determined by connecting the sulcus of the medial epicondyle and the lateral epicondyle of the femur. The horizontal line represents the horizontal axis of Picture Archiving and Communications Systems (PACS)

**Fig. 3:**
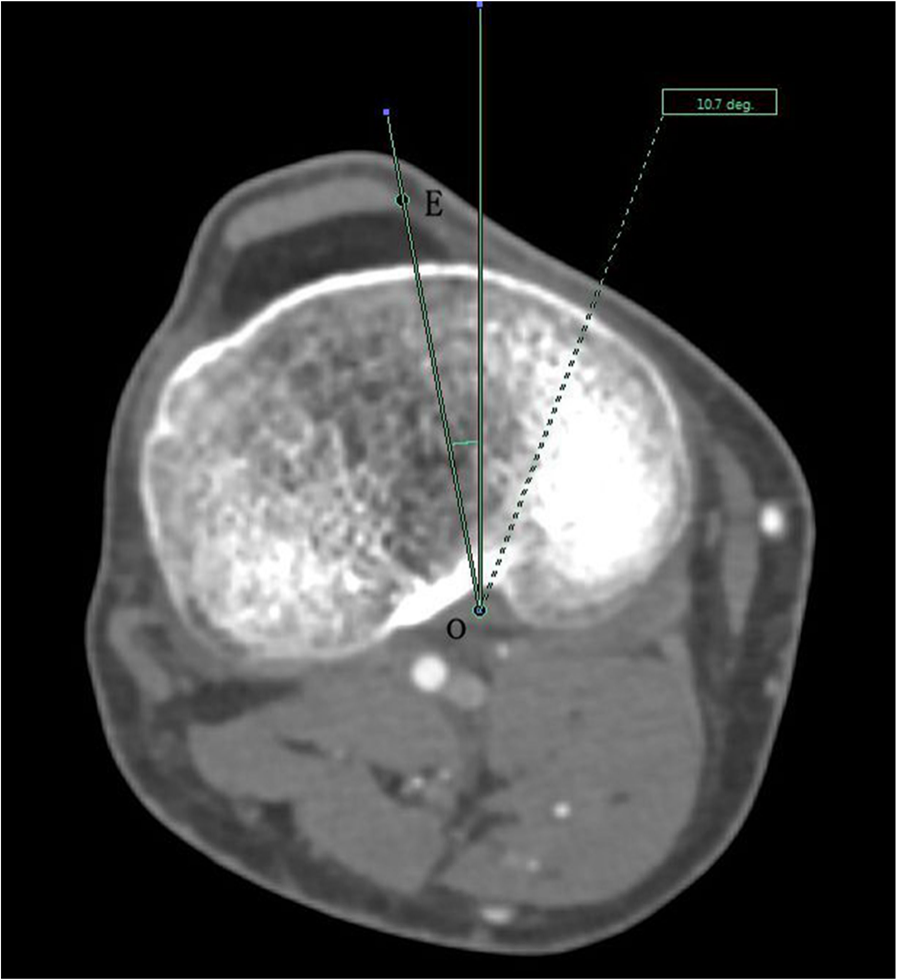
The medial edge of patellar tendon axis was drawn as a line passing through the middle of the posterior cruciate ligament (PCL) and the medial border of the patellar tendon (MBPT) at a level 8-mm below the lowest point on the lateral plateau. The PCL was recognized clearly in the posterior condylar notch of the tibia. The vertical line represents the vertical axis of PACS

**Fig. 4:**
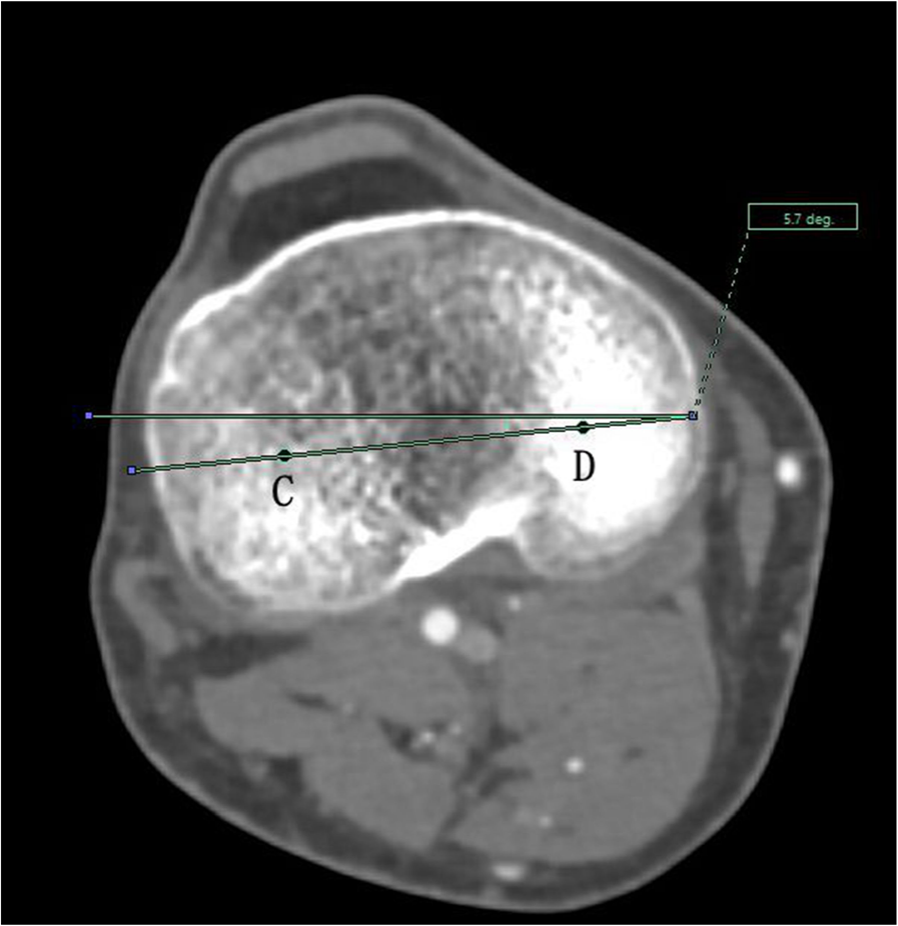
The transverse axis of the tibia is defined as the line connecting the midpoint of the medial and lateral tibial condyle at a level 8-mm below the lowest point on the lateral plateau. The horizontal line represents the horizontal axis of PACS

**Fig. 5:**
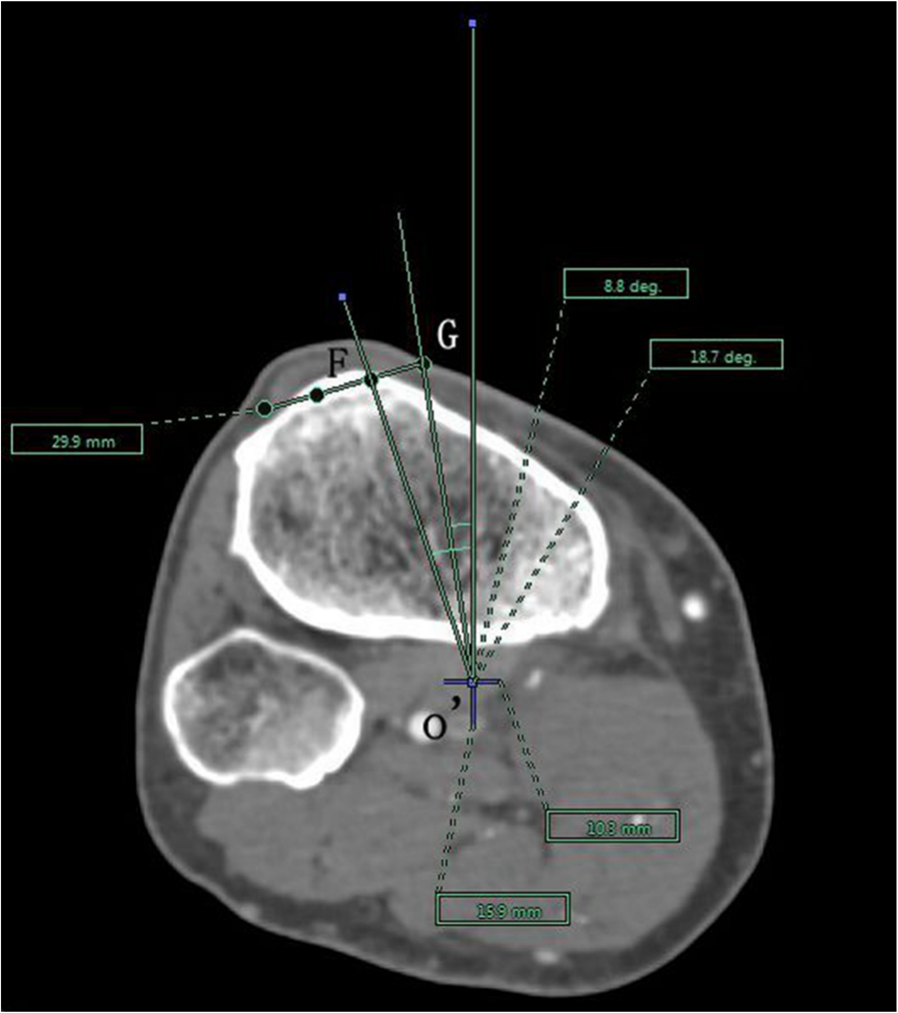
Akagi line drawn as a line passing through o’ and G; the medial 1/3 of the patellar tendon axis drawn as a line passing through o’ and F. The vertical line represents the vertical axis of PACS. o’: the projected midpoint of the PCL; G: the medial edge of the patellar tendon attachment; and F: the medial 1/3 of the patellar tendon at the level of the patellar tendon attachment

**Fig. 6:**
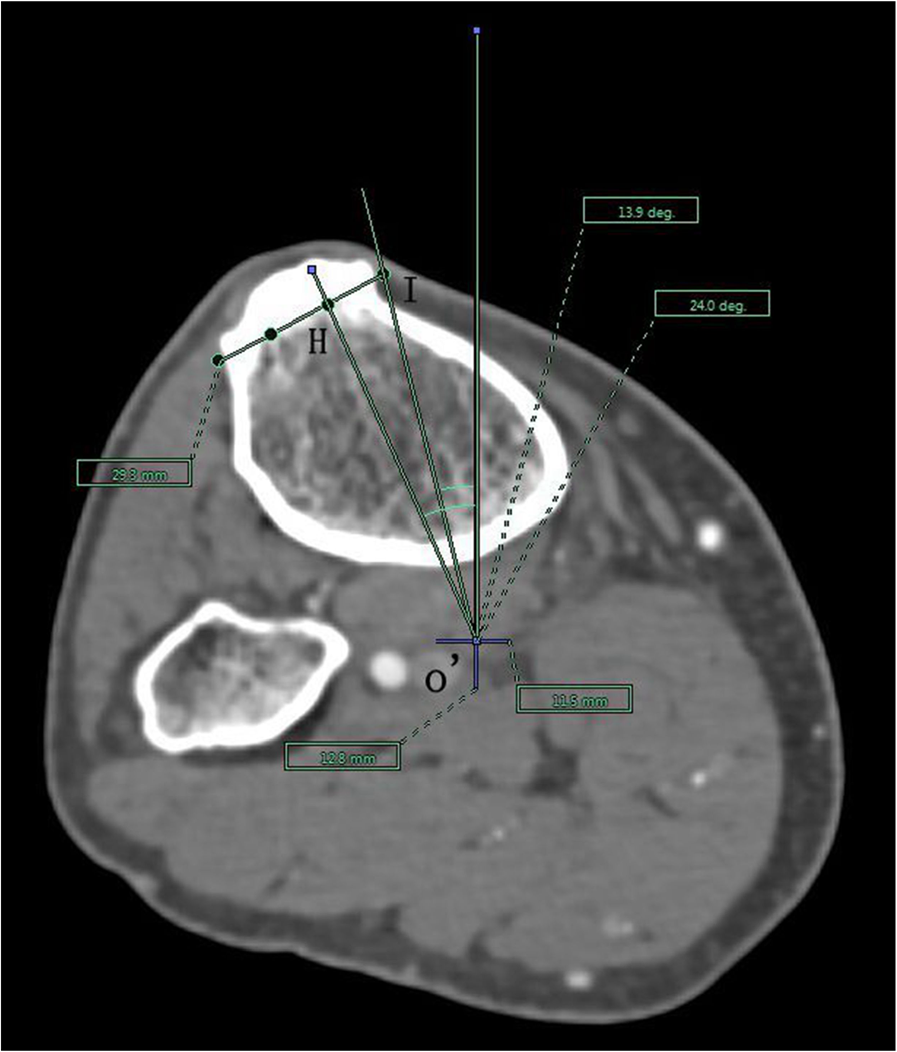
Insall line drawn as a line passing through o’ and H and medial border of tibial tubercle axis drawn as a line passing through o’ and I. The vertical line represents the vertical axis of PACS. o’: the projected midpoint of the PCL; H: the medial 1/3 of tibial tubercle, and I: medial border of tibial tubercle

**Fig. 7:**
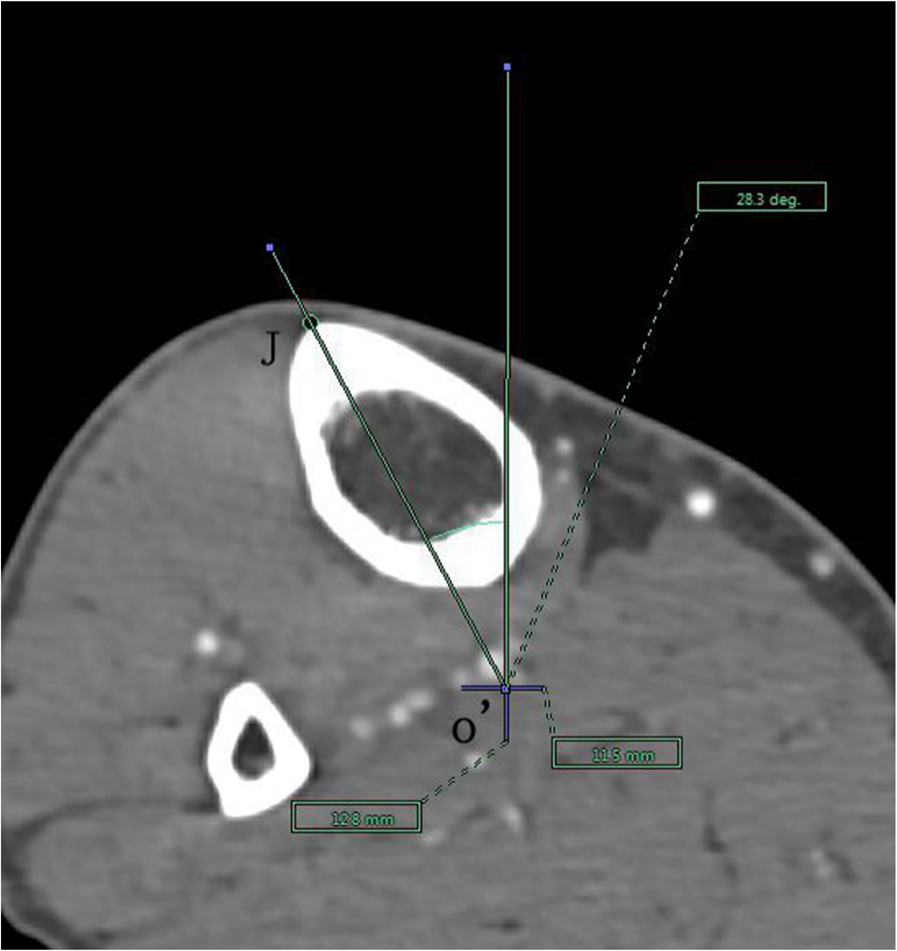
Axes of anterior tibial crest drawn as lines passing through the projected midpoint of the PCL and the 4 points(f1,f2,f3,and f4) pre-labeled on 3D reconstruction of the tibia, respectively. The vertical line represents the vertical axis of PACS. o’: the projected midpoint of the PCL; J: represents f1

**Fig. 8:**
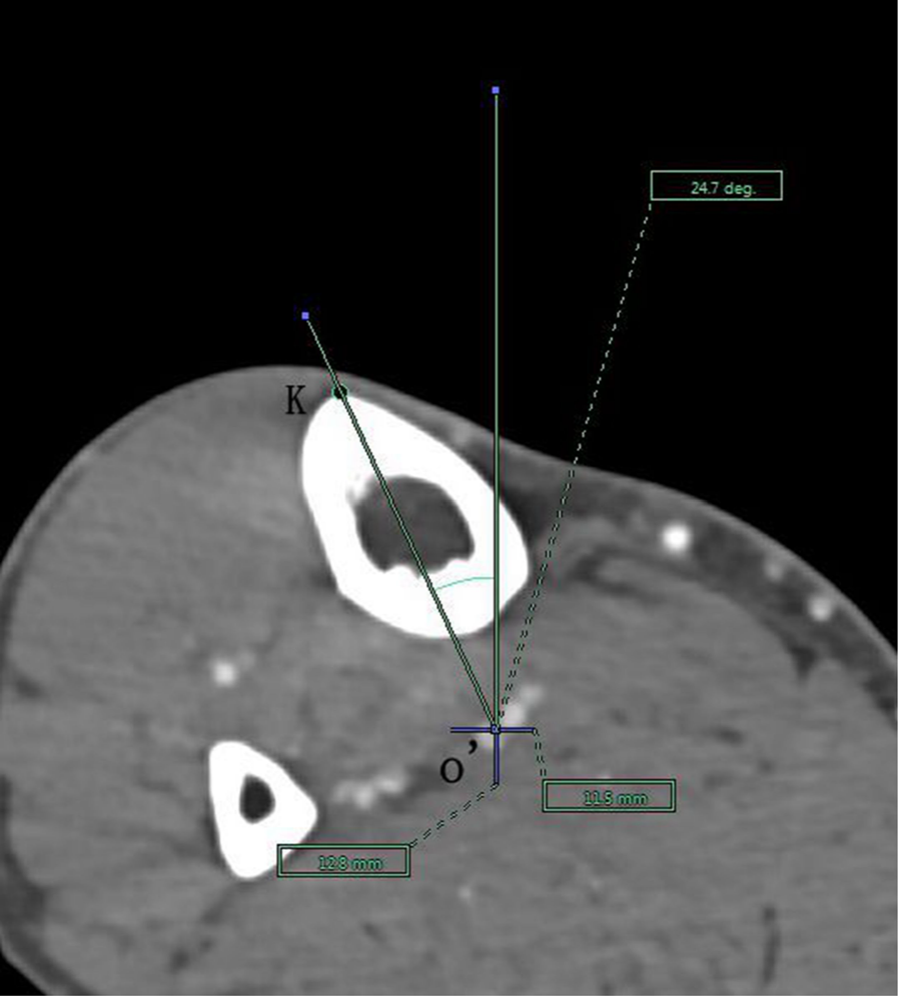
Axes of anterior tibial crest drawn as lines passing through the projected midpoint of the PCL and the 4 points(f1,f2,f3,and f4) pre-labeled on 3D reconstruction of the tibia, respectively. The vertical line represents the vertical axis of PACS. o’: the projected midpoint of the PCL; K: represents f2

**Fig. 9:**
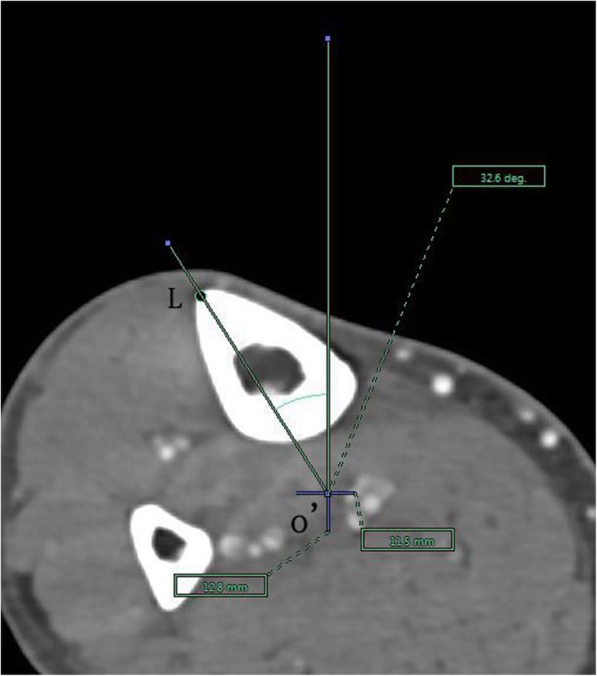
Axes of anterior tibial crest drawn as lines passing through the projected midpoint of the PCL and the 4 points(f1,f2,f3,and f4) pre-labeled on 3D reconstruction of the tibia, respectively. The vertical line represents the vertical axis of PACS. o’: the projected midpoint of the PCL; L: represents f3

**Fig. 10:**
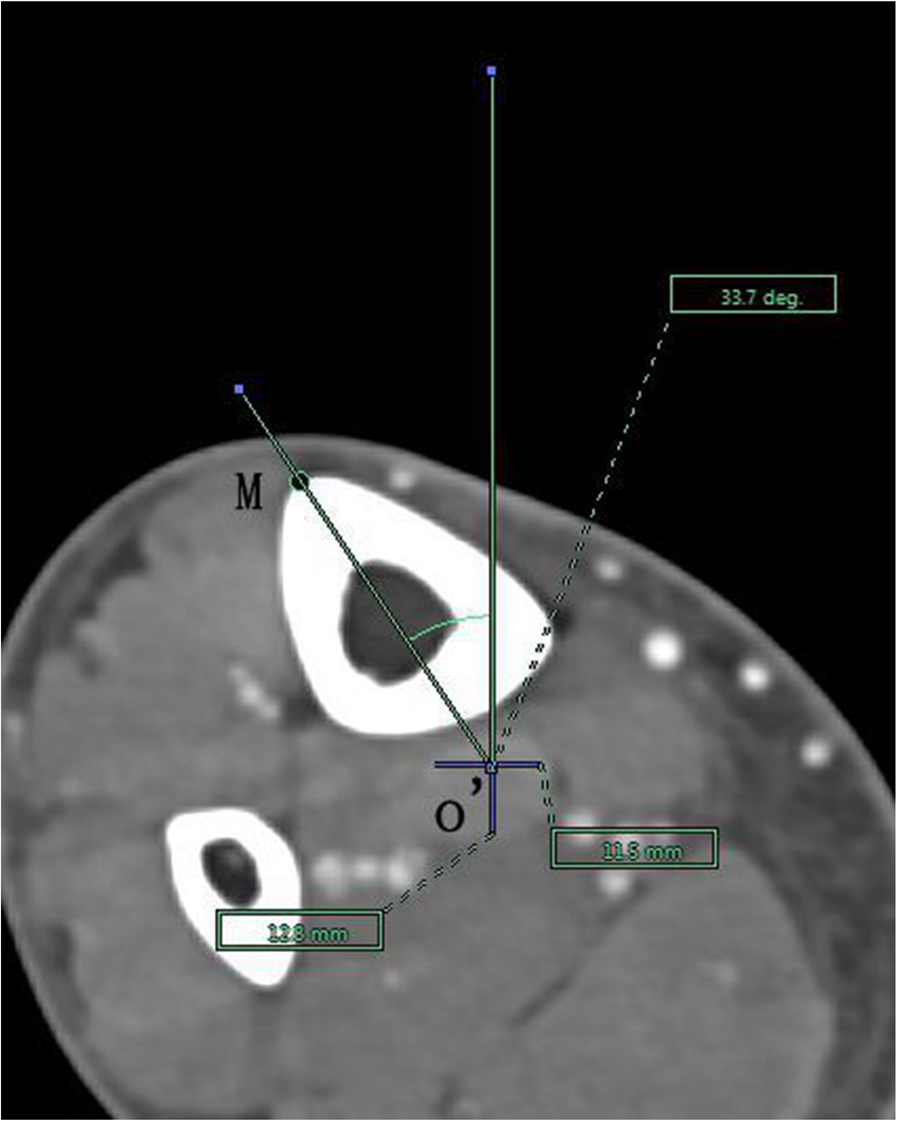
Axes of anterior tibial crest drawn as lines passing through the projected midpoint of the PCL and the 4 points(f1,f2,f3,and f4) pre-labeled on 3D reconstruction of the tibia, respectively. The vertical line represents the vertical axis of PACS. o’: the projected midpoint of the PCL; M: represents f4

The following marks and measurement were made based on the CT axis scans:

1. The angle between the PACS transverse axis and the SEA, which was determined to be connecting the most prominent points of the lateral epicondyle to the deepest point of the sulcus on the medial epicondyle of the femur (Fig. 2).
2. The angle between the PACS longitudinal axis and the line connecting the middle of the PCL and medial border of the patellar tendon (MEPT) 8-mm distal of the lateral tibial joint surface (Fig. 3).
3. The angle between the PACS transverse axis and the TAT at the level 8-mm distal to the lateral tibial joint surface (Fig. 4).
4. The angle between the PACS longitudinal axis and the line connecting the projected middle of the PCL and MEPT at the tibial attachment (Akagi line), and the angle between the PACS longitudinal axis and a line connecting the projected middle of the PCL and the medial 1/3 of the patellar tendon (M1/3) at the patellar tendon attachment level (Fig. 5).
5. The angle between the PACS longitudinal axis and the line connecting the projected middle of the PCL and MBTT and between the PACS longitudinal axis and the line connecting the projected middle of the PCL and the medial 1/3 of the tibial tubercle (Insall line) (Fig. 6).
6. The angles between the PACS longitudinal axis and the line connecting the 4 points on the anterior tibial crest (ATC1–4) and projected middle of the PCL, respectively (Figs. 7, 8, 9 and 10).

The measurement data were divided into 10 groups according to the 10 axes of the tibia, which included the Akagi line group, Insall line group, MEPT axis group, M1/3 axis group, TAT axis group, MBTT axis group, ATC1 axis group, ATC2 axis group, ATC3 axis group, and ATC4 axis group. Based on the fact that the longitudinal and transverse axes of the PACS in each scanning plane of CT were identical, the angle between SEA and TAT as well as those among the line perpendicular to SEA and the other 9 axes were measured. Compared with SEA or the line perpendicular to SEA, the external rotation was found to be positive, with negative internal rotation.

After 3 weeks, 20 randomly selected CT scans were measured again by the same author (Observer 1), while another author (Observer II) conducted an independent evaluation to determine the intra- and inter-observer variabilities. With this exercise, we derived at the following conclusion: an intraclass correlation coefficient (ICC) > 0.8 was considered to be an excellent agreement, ICC 0.6–0.8 as a fair to good agreement, and ICC < 0.6 as a poor agreement.

Quantitative data were expressed as means ± standard deviation (SD). Statistical analyses were performed using the PASW statistics 18 (SPSS Inc., Chicago, IL, USA). The normality assumption of our data was validated by the Kolmogorov-Smirnov test. Single-factor ANOVA and paired t-test were used to compare the data among the 10 axes. *P* < 0.05 was considered to be statistically significant.

## Results

The Kolmogorov–Smirnov test revealed that all data followed a normal distribution pattern. ICC and interclass correlation coefficients for the reproducibility of all parameters were > 80% (Table 1).

**Table 1:** The intraclass correlation coefficient analysis of the 11 angles of different axes relative to the longitudinal or transverse axes of Picture Archiving and Communications Systems (PACS)

	SEA	MEPT	M1/3	AL	TAT	IL	MBTT	ATC1	ATC2	ATC3	ATC4
interobserver	0.904	0.866	0.812	0.828	0.847	0.820	0.902	0.802	0.839	0.828	0.841
intraobserver	0.922	0.891	0.855	0.901	0.844	0.865	0.914	0.876	0.900	0.892	0.889

All reference axes were used for external rotation to the line perpendicular to SEA or SEA itself, except for the MPET axis, which was used for internal rotation to the line perpendicular to SEA. The Akagi line was the closest to the line perpendicular to SEA. The mean angles between the line perpendicular to SEA and Akagi line, TAT, MEPT, MBTT, M 1/3 axis, and Insall line were 1.5 ± 5.0°, 4.1 ± 5.3°, − 1.6 ± 4.5°, 3.6 ± 4.8°, 10.2 ± 5.1°, and 11.9 ± 5.4°, respectively (Table 2). The M1/3 axis and Insall line were significantly more externally rotated than the Akagi line, MEPT axis, MBTT axis, and TAT.

**Table 2:** Comparison of the angles between the line perpendicular to surgical epicondylar axis (SEA) and different tibial landmarks

Tibial axis	Mean ± SD(^°^)	Range(°)	95% confidence interval
MEPT	−1.6 ± 4.5	−12.5 ~ 15.5	−2.4 ~ −0.8
M1/3	10.2 ± 5.1	12.2 ~ 23.1	9.2 ~ 11.1
Akagi line	1.4 ± 5.0	−11.3 ~ 15.6	0.5 ~ 2.3
TAT^a^	4.1 ± 5.3	−12.4 ~ 18.3	3.1 ~ 5.0
Insall line	11.9 ± 5.4	−4.4 ~ 24.8	10.9 ~ 12.8
MBTT	3.6 ± 4.8	−11.0 ~ 15.3	2.7 ~ 4.4
ATC1	12.0 ± 6.9	−6.5 ~ 27.1	10.8 ~ 13.3
ATC2	7.2 ± 8.6	−14.6 ~ 25.8	5.6 ~ 8.7
ATC3	7.1 ± 10.4	−20.3 ~ 30.5	5.3 ~ 9.0
ATC4	6.6 ± 13.5	−22.2 ~ 35.0	4.1 ~ 9.0

^a^ the angle between SEA and TAT

**Fig. 11:**
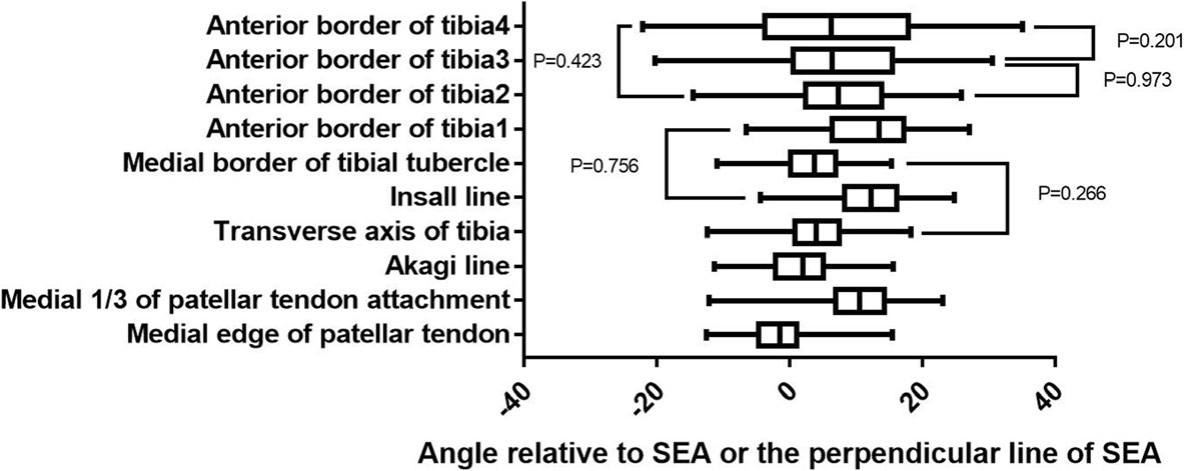
A boxplot illustrating the distributions of the angles between nine anteroposterior axes of the tibia and the line perpendicular to SEA and the angle between SEA and the transverse axis of the tibia

The mean angles between the line perpendicular to SEA and ATC1, 2, 3, and 4 axes were 12.0 ± 6.9°, 7.2 ± 8.6°, 7.1 ± 10.4°, and 6.6 ± 13.5°, respectively. The ATC 2, 3, and 4 axes were significantly more internally rotated than the M1/3 axis, Insall line, and ATC1 (Table 3). However, the ATC3 and ATC4 axes showed a greater standard deviation than the other reference markers. No significant difference was noted between the TAT and MBTT axis and among the axes of ATC2, ATC3, and ATC4 (Table 3; Fig. 11). Statistically significant differences were noted among the Akagi line, Insall line, MBTT axis, and ATC1 axis between the male and female patients (Table 4).

**Table 3:** Comparison of different tibial axes

P/t	M1/3	AL	TAT	IL	MBTT	ATC1	ATC2	ATC3	ATC4
MEPT	0.000/−25.7	0.000/−7.7	0.000/− 11.5	0.000/− 26.5	0.000/− 12.0	0.000/− 21.0	0.000/− 11.3	0.000/−9.3	0.000/− 6.7
M1/3		0.000/29.6	0.000/13.4	0.000/−5.0	0.000/22.7	0.000/−3.6	0.000/4.3	0.001/3.4	0.003/3.0
AL			0.000/−5.6	0.000/− 22.9	0.000/− 5.8	0.000/−18.3	0.000/−7.6	0.000/− 6.1	0.000/− 4.1
TAT				0.000/−16.8	0.266*/1.1	0.000/−11.7	0.000/−3.6	0.004/−2.9	0.060*/−1.8
IL					0.000/32.4	0.756*/−0.3	0.000/6.5	0.000/5.1	0.000/4.3
MBTT						0.000/−16.3	0.000/−5.1	0.000/−3.9	0.013/−2.5
ATC1							0.000/10.3	0.000/6.9	0.000/5.3
ATC2								0.973*/0.03	0.423*/0.8
ATC3									0.201*/1.2

*P>0.05

**Table 4:** Comparison of different tibial landmarks between male and female patients

Tibial axis	Mean ± SD (M)	Mean ± SD(F)	*P*-value
MEPT	−1.9 ± 4.7	−0.9 ± 3.8	0.267
M1/3	9.6 ± 5.1	11.7 ± 5.0	0.051
Akagi line	0.8 ± 5.0	3.0 ± 4.5	0.026*
TAT	3.7 ± 5.3	5.2 ± 5.2	0.164
Insall line	11.1 ± 5.2	14.0 ± 5.3	0.007*
MBTT	2.9 ± 4.5	5.3 ± 5.2	0.018*
ATC1	11.1 ± 7.0	14.5 ± 5.9	0.015*
ATC2	6.8 ± 8.8	8.3 ± 8.1	0.397
ATC3	7.7 ± 10.5	5.7 ± 9.9	0.368
ATC4	7.4 ± 13.6	4.2 ± 13.2	0.241

*P<0.05

## Discussion

Tibial rotation in TKA remains controversial. In the 1990s, Insall [16] described an anteroposterior (AP) axis from the junction of the medial and the middle-thirds of the tibial tubercle to the PCL. Wernecke et al. [20] performed MRI of 544 cases of normal knee joint and believed that Insall’s axis was a reliable landmark for rotational alignment of the tibial component, which may optimize femorotibial kinematics in fixed-bearing TKA. Until date, the Insall’s rotational axis for the tibial component in TKA has been generally accepted. Akagi [11] measured the angle between the line perpendicular to the projected SEA and a line connecting the middle of the PCL and MEPT attachment (Akagi line). He found that the mean angle between them was 0.0° ± 2.8° (range: − 6.3° − + 5.2°), which was significantly better than that between the line connecting the middle of the PCL and the medial 1/3 of the patellar tendon (10.0° ± 4.2° versus 1.6°–19.5°). Next, Akagi [10] compared the Akagi line with the transmalleolar axis and the second metatarsus bone axis in 57 healthy adults. He found that the mean angles between the line perpendicular to SEA and Akagi line was − 0.2° ± 2.8° (− 5.5°–6.3°), and those between SEA and the transmalleolar axis and second metatarsus bone axis were 25.9° ± 9° (8°–49.4°) and 5.2° ± 10° (− 21.9°–24°), respectively. Thus, the Akagi line was proven to be more reliable for determining the rotational alignment of the tibial components in TKA. Kawahara [18] believes that it is difficult to identify the center of the PCL attachment after tibial resection. He found that the axis pass the medial 1/6 of the patellar tendon at its tibial attachment and that the geometric center of tibia was useful for anterior referencing and alignment of the tibial components. Lyutzner [21] indicated that referencing the tibial rotation on a line from the medial third of the tibial tubercle to the center of the tibial tray resulted in a better femorotibial rotational alignment. Sahin [22] reported that the tibial posterior condylar line was unaffected by varus deformity, and hence could be used for guidance to determine the rotation of the tibial components. In conformance to other authors, we also found that the Akagi line was the closest one to the line perpendicular to SEA for knee joint extension. Moreover, the use of the Akagi line offered the advantages of a smaller standard deviation, relative stability, easy identification of anatomical markers, and good reproducibility. We also noted that the angle between the MBTT and the line perpendicular to SEA and that between the TAT and SEA were small, which, similar to that with the Akagi line, showed good repeatability and acted as good alignment reference markers for tibial component rotation. The Insall line was more externally rotated than Akagi line, MBTT, and TAT. Moreover, the use of the Insall line as a rotational reference for a tibial component during TKA did not show any significant adverse outcomes in several larger joint centers. This can be attributed to the fact that tibial component rotation alignment has a high tolerance for external rotation.

The anterior tibial crest is a bony hump that descends from the tibial tubercle to the anterior edge of the medial malleolus. The upper 2/3 portion is sharp, without any muscle coverage, and is extremely accessible. A previous study [23] reported that the direction of travel of the anterior tibial crest was consistent with the tibial mechanical alignment and hence could be used as a reference mark for TKA tibial osteotomy. However, until date, only a few related anatomical and clinical studies have been performed using the anterior tibial crest as a reference for TKA tibial component rotation alignment. The present research examined 122 normal, 3D-reconstructed tibias and found that the anterior tibial crest was not a stable straight line, rather it showed mild internal or external rotation. Accordingly, we selected 4 points during the study design and connected these points with the projected middle of the PCL, referring to them as ATC1, ATC2, ATC3, and ATC4 axes. The respective angles from the line perpendicular to SEA of these points were 12.0 ± 6.9°, 7.2 ± 8.6°, 7.1 ± 10.4°, and 6.6 ± 13.5°, respectively. It was observed that the external rotation of the axis gradually decreased from the proximal to the distal direction—the more the distal, the greater was the variation. The degrees of external rotations of the ATC2 and ATC3 axes relative to SEA were significantly lesser than those of the Insall line and ATC1 axis, and the stability was better than that with ATC4. A previous study [24] suggested that TKA tibial component rotation showed a good tolerance to external rotation and that its safety zone was 0–10° external rotation. Therefore, the middle anterior tibial crest is a good choice for TKA positioning of the tibial component rotation.

We identified several limitations to our study. First, the kinematics of the knee joint is extremely complex. It is a medial–pivot kinematic pattern in a healthy knee joint. In addition, SEA is not completely consistent with the flexion and extension axes of the knee joint, which has less axis rotation and greater variability post-TKA. Therefore, the use of a fixed axis as a reference for tibial component rotation alignment has some limitations. Second, a certain degree of tibial retroversion exists in TKA. The simulated tibial osteotomy plane in this study showed no retroversion, which may have led to some deviations. Third, our sample size was small. A prospective study with a larger sample size is expected to produce more accurate results with better data extrapolation. Finally, the study included only Chinese subjects residing in Northwest China. Therefore, the data can be classified as being typically east Asian knee data, which may not be applicable to the Caucasian or other populations. Therefore, the results of the present study should be interpreted cautiously.

## Conclusions

The Akagi line, MBTT, and TAT showed good consistency with SEA for axial femorotibial alignment with the knee in extension. These markers showed only a little variation and good reproducibility. The middle segment of the anterior tibial crest also demonstrated good alignment consistency with SEA in axial femorotibial alignment and hence can be used as a reliable reference marker for rotational alignment of the tibial component in TKA.

## Data Availability

The datasets used and/or analyzed in the current study are available from the corresponding authors on request.

## Abbreviations

AL: Akagi line
ATC1: Anterior tibial crest1
ATC2: Anterior tibial crest2
ATC3: Anterior tibial crest3
ATC4: Anterior tibial crest4
IL: Insall line
MBTT: Medial border of tibial tubercle
MEPT: Medial edge of patellar tendon
M1/3: Medial 1/3 of patellar tendon attachment
SEA: Surgical epicondylar axis
TAT: Transverse axis of tibia
TKA: Total knee arthroplasty
PACS: Picture archiving and communications systems
